# Supplementary figures and images for: Monitoring and characterizing soluble and membrane-bound ectonucleotidases CD73 and CD39
Source: PLoS One. 2019 Oct 25;14(10):e0220094. doi: 10.1371/journal.pone.0220094 (PMC6814236; doi:10.1371/journal.pone.0220094)

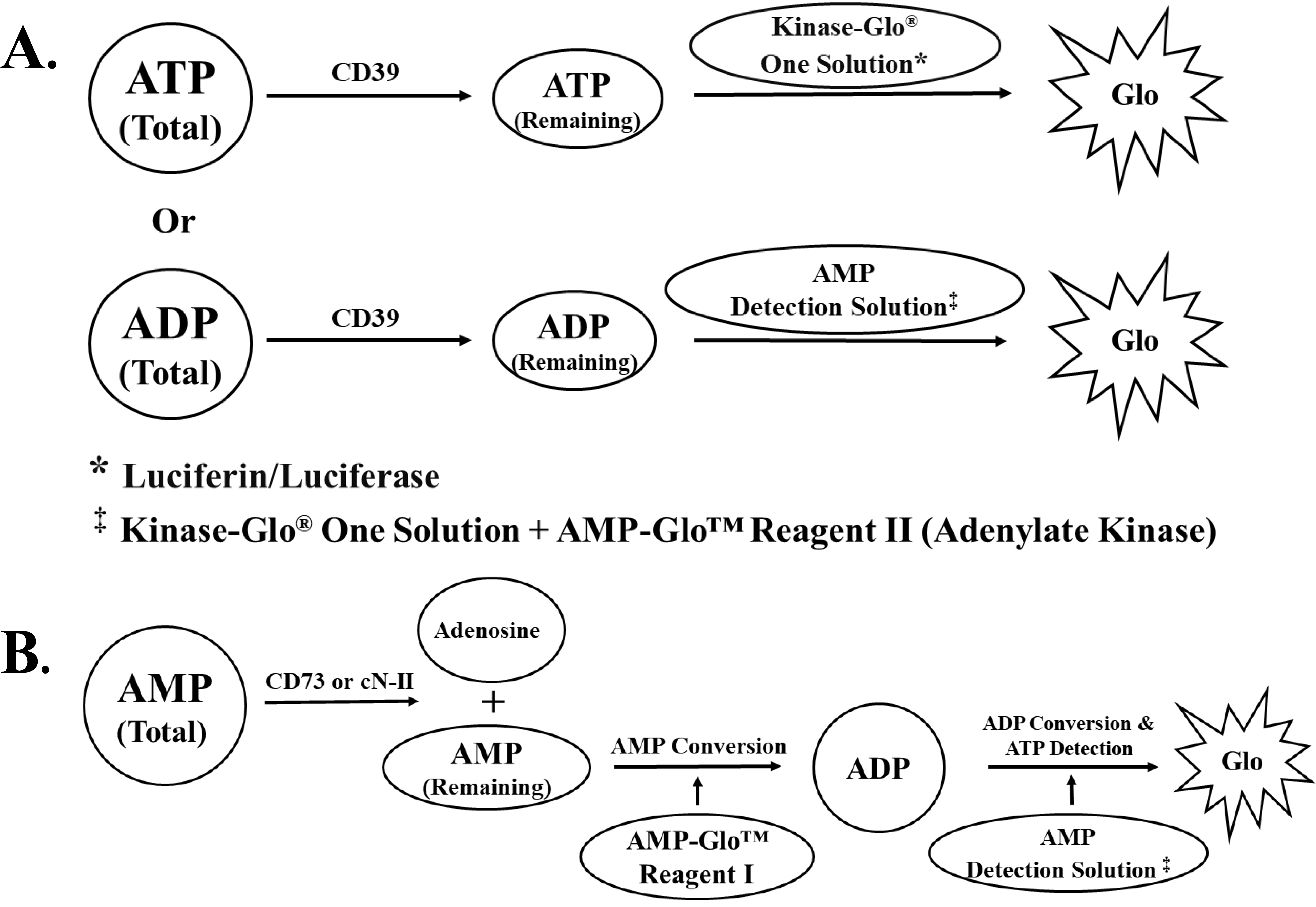

Supplement: S1 Fig — (A) Monitoring the enzyme activity of CD39 using either ATP or ADP as substrate. The principle of the assay is based on the consumption of ATP as substrate by CD39 which can be monitored by determining the amount of ATP remaining in the reaction by an ATP utilizing luciferase reaction. Alternatively, when ADP is used as a substrate, remaining ADP after CD39 reaction can be converted to ATP using adenylate kinase and the ATP generated is determined by an ATP utilizing luciferase reaction. (B) Monitoring the activity of CD73 using AMP substrate and converting remaining AMP in the reaction to ATP via two enzymes (AMP-polyphosphate phosphotransferase and adenylate kinase) and the generated ATP is detected using luciferase reaction. (TIF) [file pone.0220094.s001.tif]
