# Supplementary material for: Monitoring and characterizing soluble and membrane-bound ectonucleotidases CD73 and CD39
Source: PLoS One. 2019 Oct 25;14(10):e0220094. doi: 10.1371/journal.pone.0220094 (PMC6814236; doi:10.1371/journal.pone.0220094)
Supplement: S1 Table — (DOCX) [file pone.0220094.s002.docx]

**S1 Table: List of Antibodies tested including immunogen/Host information**

| **Antibodies (500µl/each)** |  | **Host/Isotype** | |  |  |  | **Species** | **Immunogen** |
| --- | --- | --- | --- | --- | --- | --- | --- | --- |
| #1. PA 5-11871 (Pierce) |  | Polyclonal, Rabbit/IgG, | | |  |  | Human | Recombinant fragment region within aa520-550  human CD73 |
| #2. PA 5-29750 (Pierce, 0.78mg/ml) | | Polyclonal, Rabbit/IgG, | | |  |  | Human | Recombinant fragment region within aa35-261  human CD73 |
| #3. PA 5-27336 (0.97mg/ml) |  | Polyclonal, Rabbit/IgG, | | |  |  | Human | Recombinant fragment region within aa211-538  human CD73 |
| #4. Life Tech 410200 (100µg/200µl,0.5mg/ml) | | Moise Monoclonal/IgG2a, kappa, [7G2] | | | |  | Human | Purified Human placental CD73 |
| #5. Ab 54217 (7G2, 0.5mg/ml) |  | Moise Monoclonal/IgG2a, kappa, [7G2] to CD73 | | | | | Human | Full length purified Human placental CD73 |
| #6. Ab 71322 (0.2mg/ml) |  | Polyclonal, Rabbit/IgG, | | |  |  | Human | CD73-N-terminal |
| #7. Ab 81720 (0.1mg/ml) |  | Moise Monoclonal/IgG [4G4] to CD73 | | | |  | Human | Human endothelial cell preparation from inflamed  synovial stroma from rheumatoid arthritis patients |
| #8. Millipore MABD122 (0.4mg/ml) | | Clone AA60-E3-3, Monoclonal mouse/IgG1k, | | | | | Human | DLEC (Whole Mouse Dermal Lymphatic  Endothelial Cells |
| #9. Cell Signaling (13160S) |  | Monoclonal (D7F9A) Rabbit mAb, Rabbit/IgG, | | | | | H, M, Rat | endogenous levels of total NT5E/CD73 protein |
| #10. Ab 189258 (A1, 1mg/ml, **CD39**) | | Mouse monoclonal [A1] to CD39 | | | |  | Human | Tissue, cells or virus corresponding to Human CD39.  (PHA activated Human lymphocytes). |
| #11. None: 0.1% BSA in Hank's Buffer | |  |  |  |  |  |  |  |
