# Supplementary material for: Monitoring and characterizing soluble and membrane-bound ectonucleotidases CD73 and CD39
Source: PLoS One. 2019 Oct 25;14(10):e0220094. doi: 10.1371/journal.pone.0220094 (PMC6814236; doi:10.1371/journal.pone.0220094)
Supplement: Attachment — Submitted filename: Response to Reviewers Oct 2019.docx [file pone.0220094.s004.docx]

**Reviewers comments and Response
Reviewer #1:**
1. We have clarified the legend for Fig1A to indicate that the presence or absence of ATP during determination of AMP does not affect the values of AMP measurements. This show that we can measure the concentration of AMP even in the presence of high concentrations of ATP since we remove ATP using adenylate cyclase before converting AMP to ATP with simulations inhibition of the adenylate cyclase (US Patent #20100227344 and 8183007).

2. We have also corrected the grammar and spelling errors as recommended

**Reviewer #2**

**Major**
We have added Table 1 in the supplement section which describes the immunogen and host of the antibody clones used in the study as recommended. We also replaced Fig 7 with a better resolution and higher clarity one as recommended by the reviewer.

**Minor points:**

Spelling and text mistakes have been corrected.

We also replaced the original #8 reference with Bastid et al as recommended

Ref# 8. Bastid J, Regairaz A, Bonnefoy N, Cecile D, Giustiniani J, Laheurte C, et al. Inhibition of CD39	enzymatic function at the surface of tumor Cells alleviates their immunosuppressive activity. Cancer	Immunol Res. 2015; 3:254-264

There is no duplication of references, reference#29 is not in the revised text
